# Lack of association between cigarette smoking and Epstein Barr virus reactivation in the nasopharynx in people with elevated EBV IgA antibody titres

**DOI:** 10.1186/s12885-018-4110-6

**Published:** 2018-02-14

**Authors:** Yufeng Chen, Yifei Xu, Weilin Zhao, Xue Xiao, Xiaoying Zhou, Longde Lin, Tingting Huang, Jian Liao, Yancheng Li, Xiaoyun Zeng, Guangwu Huang, Weimin Ye, Zhe Zhang

**Affiliations:** 1 Department of Otolaryngology-Head & Neck Surgery, First Affiliated Hospital of Guangxi Medical University, 6# Shuangyong Road, Nanning, Guangxi 530021 China; 2 Ministry of Education, Key Laboratory of High-Incidence-Tumor Prevention & Treatment (Guangxi Medical University), Nanning, Guangxi China; 3 Department of Epidemiology, School of Public Health, Guangxi Medical University, Nanning, Guangxi China; 4 Cancer Institute of Cangwu County, Wuzhou, Guangxi China; 5 Department of Medical Epidemiology and Biostatistics, Karolinska Institutet, Stockholm, Sweden

**Keywords:** Cigarette smoking, Epstein-Barr virus, Nasopharyngeal EBV load, Nasopharyngeal carcinoma, Nasopharyngeal EBV reactivation

## Abstract

**Background:**

Subjects with elevated Epstein-Barr virus (EBV) immunoglobulin A (IgA) titers have a higher risk of developing nasopharyngeal carcinoma (NPC), indicating that reactivation of EBV in the local mucosa might be important for NPC carcinogenesis. Cigarette smoking appears to be one of the environmental risk factors for NPC. However, it remains unclear whether smoking-induced nasopharyngeal carcinogenesis acts through reactivating EBV in the nasopharyngeal mucosa. Therefore, this study aims to investigate the association between cigarette smoking and nasopharyngeal EBV reactivation in a NPC high-risk population.

**Methods:**

A NPC high-risk cohort study, established from a population-based NPC screening program of 22,816 subjects, consisted of 1045 subjects with elevated serum IgA antibodies against EBV viral capsid antigen (VCA/IgA). Among high-risk subjects, information on detailed cigarette smoking history was collected among 313 male subjects. The associations between cigarette smoking and EBV antibody levels, EBV DNA load of the nasopharynx were analyzed.

**Results:**

No significant association was observed between either nasopharyngeal EBV DNA load or serum VCA/IgA titers and smoking status, age at smoking initiation, daily smoking intensity, smoking duration, cigarette type, or pack-years of smoking. Cigarette smoking characteristics in all subgroups did not correlate with nasopharyngeal EBV DNA positivity or EBV VCA/IgA seropositivity.

**Conclusions:**

In a population at high risk of NPC, our study suggests that cigarette smoking is neither associated with nasopharyngeal EBV DNA load nor serum VCA/IgA antibody level. Smoking-associated NPC carcinogenesis may act through other mechanisms than reactivating nasopharyngeal EBV replication.

## Background

Epstein-Barr virus (EBV) infection is a major etiologic factor for nasopharyngeal carcinoma (NPC), and EBV is detected in tumor cells of virtually all NPC cases [1]. The probability of developing NPC is 6.7–41.9 times higher for subjects seropositive for immunoglobulin A antibodies against viral capsid antigen (VCA/IgA) than those with undetectable antibodies [2].

Amongst potential non-viral causes of NPC, cigarette smoking has been intensively studied. Some early studies failed to show a link between tobacco smoking and NPC risk [3–7], while recent evidence supported a positive association between cigarette smoking and NPC risk [8–11]. Our recent population-based case-control study with a large sample size in southern China, observed that active smoking in males significantly increased the NPC risk, particularly among those heavey smokers. Moreover, exposure to passive smoking during childhood and from a spouse during adulthood was independently associated with an increased NPC risk [12]. In Taiwan, a large-scale 20-year follow-up cohort study reported that the NPC risk was closely associated with long and heavy cigarette smoking and the association persisted even when EBV seromarkers were included in the multivariate analysis [13]. In addition, cigarette smoking is an independent prognostic factor for poor NPC survival [14, 15], and is associated with a higher NPC mortality [16]. Besides epidemiological study, in vitro experiment showed that cigarette extracts could induce EBV reactivation in NPC cells [8], suggesting that cigarette smoking plays an important role in NPC carcinogenesis.

The high EBV titers in high risk NPC populations in endemic region indicate the reactivation of EBV in vivo. To date, the cell origin of reactivated EBV particles has not been addressed. Previously, we found that nasopharyngeal EBV load is correlated with serological EBV titers in high risk NPC population [17]. These clues lead us to hypothesize that cigarette smoking might be directly responsible for the reactivation of nasopharyngeal EBV and cause peripheral EBV IgA seropositivity. Thus, we investigated the association of cigarette smoking with nasopharyngeal EBV reactivation in a population based NPC high-risk cohort study.

## Methods

### Study population

The establishment of the study cohort has been described previously [17]. In brief, this study was based on a prospective NPC screening program conducted in three towns (Libu, Shatou and Shiqiao) of Cangwu County, southern China, between 2006 and 2013. Briefly, local residents ages 30–59 were enrolled in the screening program. A serum sample was taken from each subject for detection of EBV VCA/IgA antibody by immunoenzymatic assay, and each subject was offered an otorhinolaryngologic and neck lymphatic examination. A total of 22,186 individuals volunteered to take part in the initial screening program, and the participation rate was 56.2%. In total, 1045 healthy subjects seropositive with EBV VCA/IgA (VCA/IgA ≥1:5) identified from the 22,186 participants were defined as NPC high-risk population and followed up for NPC occurrence. During the follow-up period of 2010–2013, 8 individuals were diagnosed as NPC cases (5 in 2010, 3 in 2011). According to the American Joint Committee on Cancer (AJCC) TNM Staging System (7th ed., 2010), 7 of the 8 cases were early-stage NPCs (2 cases of stageI, 5 cases of stage II, and 1 case of stage III).

In 2010, a follow-up serological retest and endoscopy examination were performed in the 1045 subjects. Of them, 822 (78.4%) subjects agreed to both provide a nasopharyngeal swab and to complete a questionnaire about smoking and other lifestyle factors. Because fewer than 3% (15/509) of the participating females had ever smoked, only the 313 males were included in the analysis.

### Ethics statement

Our study group has strictly abided by the principles of Helsinki Declaration and International Ethical Guidelines for Biomedical Research Involving Human Subjects developed by the Council for International Organizations of Medical Sciences (CIOMS). The NPC screening project involved in this study was started from 2006. From 2006 to 2008, staff in Cangwu Cancer Institute conducted screening program as a government-funded health promotion project, and no biological samples were preserved. Since 2010, we joined this project and conducted etiological studies after being approved by the Ethics Review Committee of First Affiliated Hospital of Guangxi Medical University in 2009. All the human materials, serological test results, and questionnaire data were collected during the follow-up stage in 2010. Written informed consents were obtained from all participants. Any published reports involved in the study would not reveal the participants’ identification.

### Cigarette smoking assessment

Questionnaire interviews were administered by trained local health workers. Individuals who had ever smoked at least one cigarette every 1–3 days for at least 6 months were defined as ever smokers, including current smokers and ex-smokers. Ex-smokers were defined as those who had quit smoking more than 1 year before the interview. Data on age at which the individual started and stopped smoking, daily cigarettes smoked, and type of cigarettes smoked (filtered, nonfiltered, or both) were collected.

### EBV serology and DNA load measurement

Serum EBV VCA/IgA antibody levels were determined by immunoenzymatic assay as described previously [18]. Briefly, cell smears were prepared from B95–8 cultures, fixed in acetone and used in the indirect immunoenzymatic method with peroxidase-conjugated anti-human IgA antibody. Sera diluted to 1:5 were added to separate wells of slide. The slides were incubated at 37 °C for 30 min in a humid atmosphere, and washed 3 times with phosphate-buffered saline (PBS). Peroxidase-conjugated antihuman IgA antibody in appropriate dilution was added to the slides. The slides were incubated again for 30 min, washed 3 times with PBS, and flooded with diaminobenzidine and H_2_O_2_ for 10 min. Positive and negative control sera were incubated in each experiment. A serum was considered positive if the cells in the well that contained the 1:5 dilution showed brown color characteristic of this test. The blood specimens from persons antibody-positive in the initial screening were tested in further dilutions. The highest dilution of serum still positive for IgA antibody to VCA was considered as the antibody titer of that serum.

Two real-time quantitative polymerase chain reaction (qPCR) systems were set up to detect EBV DNA load and the *β-globin* gene as described previously [19, 20]. The *β-globin* gene was used as a quality control for the nasopharyngeal swab sampling, DNA extraction and PCR reaction. A standard curve of the CT values obtained from plasmid DNA containing *BamHI-W* or *β-globin* fragment respectively was established in parallel. Each sample was tested in duplicate, and the mean of the two values was taken as the copy number of the sample. Samples were defined as negative if the CT values exceeded 40 cycles. In all experiments appropriate negative and positive controls were included during nucleic acid isolation and amplification. Swab DNA samples were renumbered before EBV DNA load detection to ensure a blind test. The copy numbers of EBV DNA or *β-globin* gene per swab (expressed in copies/swab) were calculated according to the following equation:

$$ C=Q\times \frac{V_{DNA}}{V_{PCR}}\times \frac{1}{V_{EXT}} $$

C: target concentration in one swab (copies/swab).

Q: target quantity (copies) determined by PCR.

V_DNA_: total volume of DNA obtained after extraction (50ul).

V_PCR_: volume of DNA solution used for PCR (1ul).

V_EXT_: volume of saline solution extracted (1 swab).

### Statistical analysis

The SPSS statistical analysis software (Version 16.0, SPSS Inc., Chicago, IL) was used for all statistical analyses. Since the EBV DNA load and serum VCA/IgA antibody titers were non-normal distribution data, we conducted a log-transformed (log10-transformed) to obtain approximately normal distribution of the variables. To compare demographics, EBV DNA load and VCA/IgA antibody titers between different smoking subgroups, we used Chi-square test for categorical variables and one-way ANOVA for continuous variables. Linear trend tests for associations between smoking subgroups and EBV DNA load and serum VCA/IgA antibody titers were performed for ordinal variables. Multivariate unconditional logistic regression models were used to estimate odds ratios (ORs) and corresponding 95% confidence intervals (CIs) for associations between cigarette smoking and nasopharyngeal EBV DNA status or serum VCA/IgA status. All statistical tests were two-sided and a *P* value of < 0.05 was considered statistically significant.

## Results

### Study population characteristics

Demographic characteristics of the 313 males seropositive for VCA/IgA, stratified by smoking status, are shown in Table 1. Among them 75.4% (236/313) were current smokers, while former smokers and never smokers accounted for 12.8% (40/313) and 11.8% (37/313), with mean ages of 49.7, 49.5, and 47.3 years, respectively. Subjects with an ever smoking history were more likely to reside in Libu and Shatou than in Shiqiao, while the distribution of age and education level had no statistical difference among the three smoking groups (*P* > 0.05).

**Table 1:** Characteristics of male nasopharyngeal carcinoma high-risk population by smoking status, Cangwu county, China, 2006–2013

Characteristics	Smoking status						*P* Value^a^
	Never (*N* = 37)		Former (*N* = 40)		Current (*N* = 236)		
	No.	%	No.	%	No.	%	
Residential area							0.039
Libu	4	11.1	2	5.6	30	83.3	
Shatou	9	6.5	21	15.1	109	78.4	
Shiqiao	24	17.4	17	12.3	97	70.3	
Age							0.176
Mean(SD)	47.3(7.5)		49.5(9.3)		49.7(8.0)		0.323
30–39	4	10.0	6	15.0	30	75.0	
40–49	19	18.3	12	11.5	73	70.2	
50–59	14	8.3	22	13.1	133	78.7	
Education level							0.329
Primary school or below	16	10.1	20	12.6	123	77.4	
Secondary school	15	13.6	18	16.4	77	70.0	
High school or above	6	13.6	2	4.5	36	81.8	

^a^*P* value for the comparison of means of age was determined by a one-way ANOVA, other *P* values were determined by a chi-square test. Abbreviation: SD, standard deviation

### Associations between cigarette smoking and nasopharyngeal EBV DNA load, serum VCA/IgA titers

We found no significant associations between nasopharyngeal EBV DNA load and cigarette smoking characteristics, including smoking status, age at smoking initiation, number of cigarettes smoked per day, smoking duration, type of cigarettes and pack-years of smoking (*P* > 0.05, Table 2). Cigarette smoking also was not associated with serum VCA/IgA titers (*P* > 0.05 for all subgroups, Table 2).

**Table 2:** Relationship between smoking and nasopharyngeal EBV load, EBV VCA/IgA antibody

Smoking characteristics	No.	EBV DNA copies, log_10_ (Mean ± SD)	*P* value^a^	VCA/IgA titer, log_10_ (Mean ± SD)	*P* value^a^
Smoking status					
Never smoker	37	2.87 ± 1.60	0.384	1.05 ± 0.21	0.228
Former smoker	40	3.36 ± 1.55		1.00 ± 0.21	
Current smoker	236	3.19 ± 1.58		1.07 ± 0.23	
*P*_trend_			0.251		0.551
Age at smoking initiation (years)					
< 20	127	3.05 ± 1.68	0.123	1.07 ± 0.22	0.924
20–29	123	3.29 ± 1.53		1.06 ± 0.24	
≥30	26	3.71 ± 1.10		1.04 ± 0.24	
* P*_trend_			0.025		0.911
Smoking intensity (cigarettes/day)					
≤10	51	3.27 ± 1.47	0.604	1.06 ± 0.23	0.282
11–30	159	3.19 ± 1.62		1.07 ± 0.23	
> 30	31	3.33 ± 1.49		1.00 ± 0.20	
* P*_trend_			0.273		0.327
Smoking duration (years)					
≤15	24	3.23 ± 1.54	0.551	0.99 ± 0.24	0.350
16–30	123	3.14 ± 1.59		1.06 ± 0.22	
> 30	129	3.29 ± 1.58		1.08 ± 0.23	
* P*_trend_			0.224		0.240
Type of cigarettes smoked					
Filtered cigarettes	174	3.23 ± 1.56	0.635	1.07 ± 0.23	0.895
Non-filtered cigarettes	34	3.26 ± 1.35		1.08 ± 0.24	
Both type	68	3.15 ± 1.72		1.05 ± 0.22	
* P*_trend_			0.392		0.926
Cumulative smoking (pack-years^b^)					
< 20	95	3.16 ± 1.53	0.202	1.03 ± 0.23	0.252
20–39	136	3.37 ± 1.59		1.09 ± 0.24	
≥40	45	2.91 ± 1.58		1.04 ± 0.19	
* P*_trend_			0.768		0.846

^a^Comparisons were performed using one-way ANOVA; ^b^ pack-years = (number of cigarettes smoked per day/20) × number of years smoked. Abbreviation: EBV, Epstein-Barr virus; VCA/IgA, IgA antibodies against viral capsid antigen; SD, standard deviation

As shown in Tables 3, 87% (272/313) of male high-risk individuals was positive for nasopharyngeal EBV DNA, and 13% (41/313) was EBV DNA negative. In the follow-up serological retest, 7% (23/313) subjects’ VCA/IgA antibodies turned to be negative, and because of the small number of seronegative subjects we classified VCA/IgA negative and titer of 1:5 subjects into one group (30%, 95/313), thus the rest was another group with VCA/IgA ≥ 1:10 (70%, 218/313). Cigarette smoking variables in all aspects did not show any positive association either with nasopharyngeal EBV DNA status or serum VCA/IgA status (Table 3).

**Table 3:** The association between cigarette smoking and nasopharyngeal EBV DNA positivity, serum VCA/IgA antibody status

Variable	Nasopharyngeal EBV DNA				VCA/IgA titer			
	Negative	Positive	OR^a^ (95% CI)	OR^b^ (95% CI)	Negative or 1:5	≥ 1:10	OR^a^ (95% CI)	OR^c^ (95% CI)
Smoking status								
Never smoker^d^	6	31	1.00 (reference)	1.00 (reference)	11	26	1.00 (reference)	1.00 (reference)
Former smoker	4	36	1.28 (0.49–3.32)	1.34 (0.51–3.54)	12	28	0.96 (0.45–2.06)	0.72 (0.32–1.58)
Current smoker	31	205	1.74 (0.45–6.74)	1.73 (0.43–6.69)	72	164	0.99 (0.37–2.62)	0.76 (0.27–2.09)
Age at smoking initiation (years)								
< 20	20	107	1.04 (0.38–2.80)	1.06 (0.38–2.95)	41	86	0.89 (0.40–1.97)	0.64 (0.28–1.49)
≥20	15	134	1.73 (0.62–4.82)	1.79 (0.63–5.07)	43	106	1.04 (0.47–2.30)	0.79 (0.35–1.81)
Smoking intensity (cigarettes/day)								
≤10	6	45	1.60 (0.46–5.58)	1.75 (0.50–6.21)	20	31	0.61 (0.24–1.56)	0.54 (0.21–1.39)
11–30	27	167	1.19 (0.44–3.21)	1.20 (0.44–3.24)	51	143	1.01 (0.45–2.25)	0.92 (0.41–2.08)
> 30	2	29	2.91(0.53–15.80)	3.08 (0.56–16.85)	13	18	0.51 (0.18–1.43)	0.45 (0.16–1.29)
Smoking duration (years)								
≤15	3	21	1.36 (0.31–6.03)	2.12(0.42–10.80)	9	15	0.71 (0.24–2.09)	0.80 (0.24–2.68)
16–30	17	106	1.21 (0.44–3.32)	1.37 (0.49–3.84)	42	81	0.82 (0.37–1.81)	0.71 (0.31–1.64)
> 30	15	114	1.47 (0.53–4.11)	1.20 (0.36–3.93)	33	96	1.23 (0.55–2.76)	0.71 (0.28–1.79)
Type of cigarettes smoked								
filtered cigarettes	21	153	1.41 (0.53–3.78)	1.55 (0.57–4.23)	48	126	1.11 (0.51–2.42)	2.32 (0.94–5.75)
non-filtered cigarettes	3	31	2.00 (0.46–8.72)	1.52 (0.33–7.05)	9	25	1.18 (0.42–3.32)	2.30 (0.88–4.39)
both type	11	57	1.00 (0.38–2.97)	0.97 (0.31–3.01)	27	41	0.64 (0.27–1.51)	1.90 (0.72–4.81)
Cumulative smoking (pack-years)								
< 20	13	82	1.22 (0.43–3.50)	1.03 (0.30–2.58)	35	60	0.73 (0.32–1.65)	0.64 (0.27–1.50)
20–39	15	121	1.56 (0.56–4.35)	1.44 (0.49–4.27)	35	101	1.22 (0.55–2.73)	0.83 (0.36–1.95)
≥40	7	38	1.05 (0.32–3.45)	1.59 (0.59–4.29)	14	31	0.94 (0.36–2.41)	0.68 (0.25–1.86)

^a^The OR without adjustment; ^b^ OR adjusted for residential area, age, education level, VCA/IgA antibody titers; ^c^ OR adjusted for residential area, age, education level, nasopharyngeal EBV DNA load; ^d^ Never smokers were the reference group for all comparisons. Abbreviation: EBV, Epstein-Barr virus; VCA/IgA, IgA antibodies against viral capsid antigen; CI, confidence interval

## Discussion

Epidemiologic studies have demonstrated cigarette smoking is associated with excess NPC risk. In vitro experiments also found that cigarette extracts promote EBV replication in B cells and NPC cells [8]. Therefore, it has been proposed that cigarette smoking may play a role in NPC development and induction of EBV reactivation. However, whether cigarette smoking directly induces EBV replication in the nasopharynx and causes EBV IgA seropositivity is unknown. In this population-based study, we found no evidence of a positive association between cigarette smoking and the EBV DNA load in the nasopharynx or with serum EBV VCA/IgA antibody titers. Our results suggest that cigarette smoking does not directly affect EBV reactivation in the nasopharynx. Thus, cigarette-smoking-associated NPC carcinogenesis might act through other mechanisms.

Except IgA antibodies of mucosa origin, smoking also induces IgG antibodies against EBV replication. For instance, cigarette smoking has been associated with titers of VCA/IgG [21], which is in turn associated with an increased risk of EBV-positive Hodgkin lymphoma [22]. These findings, combined with ours, indicate that rather than being specific to the nasopharyngeal mucosa, smoking-induced activation of EBV might be a systemic effect.

On the other hand, serologic responses to EBV, as determined by antibody levels in circulation, were used as a proxy for evaluation of the degree of EBV exposure in previous studies. Elevated serum levels of antibodies against EBV viral capsid antigen and EBV DNase may reflect the reactivation of EBV in human body [13]. The antibody production has underlying host exogenous determinants such as genetic susceptibility or the subtype of EBV [23]. What is more, other etiologic factors may also be involved as confounders. Cigarette smoking is associated with lower socioeconomic status in many populations. In addition, socioeconomic and race/ethnic differences were demonstrated in the seroprevalence of EBV [24]. Thus, in EBV related diseases, EBV antibody seropositivity, host genetic background and the habit of smoking might not act independently. Instead, they might have combined or synergistic effects on the etiology. The complicated synergistic effects result in conflicting conclusions as regard to the association between smoking, EBV seropositivity and cancer risks.

Compared to the lower aero-digestive tracts, the nasopharynx is a site which is more directly exposed to cigarette smoking. However, the magnitude of excess risks associated with smoking in cancer of the nasopharynx (OR less than 2) [8–10, 12, 25] is much lower than that for cancers of the lung and larynx (OR~ 20) [26], indicating that the nasopharyngeal epithelium might not be sensitive target cells of carcinogens from tobacco smoking. Furthermore, previous studies did not find any significant interaction between anti-EBV seromarkers and cigarette smoking for NPC development [13, 27]. These observations are plausibly consistent with our current results, indicating that the induction of NPC carcinogenesis might not be a synergistic effect of carcinogens from tobacco smoking and EBV reactivation.

Some other factors might be involved in the reactivation of nasopharyngeal EBV. The nasopharyngeal cavity is an ideal ecological niche with suitable conditions for colonization of micro-organisms which normally inhabit the nasopharynx. Some bacteria can produce short chain fatty acids such as butyric acid, which is known as an inducer of EBV replication, suggesting that microflora could be a risk factor in the development of NPC by effecting on the lytic cycle of EBV. Interestingly, one study shows that microbial communities in the upper respiratory tract can be distorted by cigarette smoking [28], thus it may be one of the potential mechanisms for cigarette-smoking-related NPC carcinogenesis, as observed by other studies [29, 30].

The advantage of this study is that it is the first population based study to investigate the association between cigarette smoking and EBV reactivation, by assessing nasopharyngeal EBV DNA load or serum VCA-IgA antibody level in an EBV seropositive high-risk population. However, there are also limitations. First, our major concern is the self-reported nature of smoking information, which might be misclassified by recall bias. Second, the influence of passive smoking on EBV load and VCA/IgA titers was not assessed as we did not collect such information. Finally, although the 1045 high-risk subjects were enrolled from a large sample size of 22,186, the number of study subjects (313 high-risk males) was relatively small.

## Conclusions

We found null association between either nasopharyngeal EBV DNA load or serum VCA/IgA titers and cigarette smoking. Cigarette smoking-associated NPC carcinogenesis might not act through reactivating nasopharyngeal EBV replication, but by other mechanisms. Our study leads to a better understanding of the etiologic interactions between viral and environmental factors in the pathogenesis of NPC.

## Abbreviations

EBV: Epstein-Barr virus
IgA: Immunoglobulin A
NPC: Nasopharyngeal carcinoma
VCA: Viral capsid antigen
